# Improving the Measurement of Functional Somatic Symptoms With Item Response Theory

**DOI:** 10.1177/1073191120947153

**Published:** 2020-08-06

**Authors:** Angélica Acevedo-Mesa, Jorge Nunes Tendeiro, Annelieke Roest, Judith G. M. Rosmalen, Rei Monden

**Affiliations:** 1 University of Groningen, University Medical Center Groningen, Interdisciplinary Center Psychopathology and Emotion regulation (ICPE), Groningen, the Netherlands; 2 University of Groningen, Department of Psychometrics and Statistics, Groningen, the Netherlands; 3 University of Groningen, Department of Developmental Psychology, Interdisciplinary Center Psychopathology and Emotion regulation (ICPE), Groningen, the Netherlands

**Keywords:** functional somatic symptoms, item response theory, graded response model, SCL-90, medically unexplained symptoms

## Abstract

More than 40 questionnaires have been developed to assess functional somatic symptoms (FSS), but there are several methodological issues regarding the measurement of FSS. We aimed to identify which items of the somatization subscale of the Symptom Checklist–90 (SCL-90) are more informative and discriminative between persons at different levels of severity of FSS. To this end, item response theory was applied to the somatization scale of the SCL-90, collected from a sample of 82,740 adult participants without somatic conditions in the Lifelines Cohort Study. Sensitivity analyses were performed with all the participants who completed the somatization scale. Both analyses showed that Items 11 “feeling weak physically” and 12 “heavy feelings in arms or legs” were the most discriminative and informative to measure severity levels of FSS, regardless of somatic conditions. Clinicians and researchers may pay extra attention to these symptoms to augment the assessment of FSS.

A symptom is defined as a bodily sensation or mental experience subjectively perceived as a change from normal health (Rhodes & Watson, 1987). Around 33% of the physical symptoms reported during consultations in primary care remain unexplained by an organic pathology (Kroenke, 2003). Such physical symptoms are called functional somatic symptoms (FSS) or medically unexplained symptoms. Although the burden of FSS on patients and the health care system is recognized (Zonneveld et al., 2013), the research and treatment of FSS are complicated partly due to the difficulty of measuring FSS.

Several problems exist regarding the measurement of FSS. First, there are more than 40 questionnaires developed to assess self-reported FSS (van Driel et al., 2017; Zijlema et al., 2013). These differ greatly in crucial aspects, such as the number and type of physical symptoms to be included as items in questionnaires, as well as the scaling of these items (e.g., Likert vs. dichotomous), which often hampers the comparison of results between studies. Second, the items used to construct FSS questionnaires have traditionally been selected based on experts’ knowledge (Zijlema et al., 2013) and although the validity and reliability of these questionnaires have been studied, it is unknown which of the individual items are most relevant to measure FSS from a more data-driven approach. Third, the severity of FSS has often been measured by sum scores of either a total count of symptoms or the addition of the severity of individual symptoms (van Driel et al., 2017), assuming that the greater the sum score, the higher the severity of FSS. However, this assumption is questionable. For instance, within the same sum-score group, one person might have several mild symptoms, whereas another might have only one very severe symptom. Thus, studying or treating these heterogeneous patients in one group may be suboptimal. Moreover, the cut-off scores used to classify patients by the severity of FSS vary among questionnaires and are based on the sum score of each questionnaire (Zijlema et al., 2013). This disguises the individuals’ severity of FSS since it ignores the specific types and frequencies of the symptoms reported individually. These methodological issues are problematic since, in research, conclusive results cannot be achieved without accurate assessments (Rief et al., 2017).

An approach to deal with these issues may consist of focusing on how specific items contribute to the measurement of FSS, instead of focusing on sum scores. This can be done by applying item response theory (IRT). IRT is a framework of statistical models that aims to obtain knowledge about a latent construct (e.g., FSS), by modeling the association between such construct and each item of a questionnaire (e.g. a symptom of FSS) through the response patterns of persons to a set of items (Reise & Waller, 2009). IRT models are based on both item parameters (e.g., item discrimination and item location or thresholds) and person parameters (person location). The item discrimination parameter (denoted α), is a slope parameter that reflects how the item distinguishes between varying levels of the latent trait scale. The item threshold parameters (denoted β) reflect the location of the item on the latent trait scale, that is, how severe a symptom is. The person location (denoted θ) reflects the person’s location on the latent trait scale (Embretson & Reise, 2013). IRT models allow relating symptom and person severity by locating items and persons on the same latent trait scale (e.g., the severity of FSS).

When applied to the construct of the severity of FSS, IRT models provide information about which items are more able to discriminate between persons with different levels of severity of FSS, as well as which items represent higher or lower severity of FSS based on their probability of being reported. This is especially relevant for research on FSS due to the heterogeneity of symptoms and the difficulty to measure the severity of FSS. Thus, identifying the discrimination abilities and the severity of each item is useful to improve the measurement of FSS.

This study aimed to identify items that can best reflect and discriminate between different severity levels of FSS. To this end, we fitted the graded response model (GRM; Samejima, 1969) to the somatization scale of the Symptom Checklist–90 (SCL-90; Derogatis, 1994), which is shown to be one of the most suitable measures for large scale studies of FSS (Zijlema et al.,2013), in a sample of adults without somatic conditions from the Lifelines population-based cohort. The GRM is an IRT model suitable to analyze items with more than two ordered response categories (e.g., Likert-type scales; Samejima, 1969). Two studies have fitted the GRM to the somatization scale of the SCL-90, in patients with neuromusculoskeletal diagnoses (D. L. Hart et al., 2012) and psychiatric disorders (Paap et al., 2011). Although these studies contributed to improve our understanding of the SCL-90, they were performed in patients with specific physical or psychiatric disorders and not in a general population. Given that around 33% of the consultations in primary care are related to FSS (Kroenke, 2003), studying the properties of the somatization scale of the SCL-90 in the general population is of relevance. Therefore, we aimed to explore the properties of items in a healthy general population sample whose symptoms are presumably unexplained by somatic conditions. Our data-driven approach could provide useful information about which items are the most relevant to measure FSS.

## Method

### Participants

For this study, we employed data from the participants of the Lifelines Cohort Study. Lifelines is a multidisciplinary prospective population-based cohort study examining in a unique three-generation design the health and health-related behaviors of 167,729 persons, including children and adults, living in the North of the Netherlands. It employs a broad range of investigative procedures in assessing the biomedical, sociodemographic, behavioral, physical, and psychological factors that contribute to the health and disease of the general population, with a special focus on multimorbidity and complex genetics (Stolk et al., 2008). Participants were excluded from the study if they had severe psychiatric or physical illnesses, limited life expectancy (<5 years), or insufficient knowledge of the Dutch language. The detailed sampling procedure can be found elsewhere (Scholtens et al., 2014). In our study, we analyzed the baseline data from the adult participants (age 18+) of the cohort, which consists of 152,928 participants.

### Sample Selection

Of the 152,928 adult participants, we excluded 4,324 who did not complete the somatization scale of the SCL-90, and 65,864 who reported a lifetime diagnosis of one or more somatic conditions. A sample of 82,740 participants was selected for analysis. We excluded participants who reported having somatic conditions to minimize the chance of symptom reporting due to an underlying pathology related to such conditions. The somatic conditions to exclude were selected based on the ranking of the most prevalent disorders per year in the Dutch population (www.volksgezondheidenzorg.info/ranglijsten; Gijsen et al., 2013). Participants with the following disorders were excluded: arrhythmia, heart attack, heart failure, diabetes Type 1 or 2, stroke, osteoarthritis, chronic obstructive pulmonary disease, dementia, rheumatoid arthritis, asthma, any type of cancer, Parkinson’s disease, epilepsy, disturbed kidney function, migraine, and osteoporosis (see Appendix A in the online supplemental material). Although the report of symptoms could still be due to somatic conditions that we did not exclude, we expect that by excluding the most prevalent somatic conditions, we have been able to achieve a presumably healthy sample.

### Measures

#### Functional Somatic Symptoms

FSS were assessed with the somatization scale of the SCL-90 (Derogatis, 1994). The scale assesses to what extent the participant was hampered by 12 different somatic symptoms in the past 7 days on a 5-point Likert-type scale from 0 (*not at all*) to 4 (*extremely*). Higher sum scores reflect a higher severity of FSS. Due to the translation to Dutch language, Item 11 “feeling weak physically” is slightly different from the original SCL-90 item, “feeling weak in parts of your body.”

### Statistical Analysis

#### The Graded Response Model Fitting

The GRM was applied to the 12 items of the somatization scale of the SCL-90. First, the following parameters were estimated with the “grm” function of the “ltm” package (D = 1) in R (Rizopoulos, 2006): (a) The discrimination parameter of each item (α). The higher the discrimination, the higher the accuracy of an item to distinguish accurately between different levels of severity of FSS. (b) Four threshold parameters for each item (β). Each threshold parameter represents the latent trait score at which persons have a 50% probability of responding above a particular response category (e.g., probability of responding in or above the option “not at all” in the item “headache”). (c) The person location (θ), which indicates the level of severity of FSS for each person. This is a standardized score with mean 0 and standard deviation 1 (Embretson & Reise, 2013). Note that item and person parameters are scaled in the same metric (Rizopoulos, 2006). An advantage of the GRM is that it handles missing data on the item level; therefore, data imputation was not necessary (Embretson & Reise, 2013).

Second, we computed the mean of the threshold parameters for each item, to obtain a mean location per item. With this information, we could order the items from low thresholds (i.e., frequently reported) to high thresholds (i.e., less frequently reported), indicating low to high levels of severity of FSS.

Third, we calculated the category response curves (CRCs) based on the parameters estimated with the GRM. These represent the probability of responding in each response category; depending on the person’s level of severity of FSS (θ). Five CRCs were calculated for each of the 12 items of the scale, each one representing the probability of answering to an item response category. As the α parameter increases, the CRCs become more peaked and narrower, indicating that the response categories are able to differentiate among trait levels appropriately. We plotted the CRCs for each item against the distribution of the θ parameter, which allows visualizing the probability of responding at each answer option and the corresponding level of severity of FSS.

Finally, we calculated the item information functions (IIFs) and the test information function (TIF). In the GRM, the concept of reliability can be replaced by item and test information (Reise & Waller, 2009). It is not assumed that the same standard error (*SE*) of measurement applies to all scores in a population, as is commonly the case in the classical test theory. Instead, the *SE* can vary across the severity of FSS. The smaller the *SE* at a specific person location θ, the higher the precision of the estimated parameter, and thus, the higher the IIFs and the TIF (Embretson & Reise, 2013). The IIFs are calculated per item, showing the precision of each item, and the TIF is calculated by adding all the IFFs, showing the precision of the scale. The IIFs and the TIF were plotted against the distribution of the θ parameter to show the location at which the item and test information is higher or lower, depending on the level of severity of FSS. All the plots were generated by using the “ggplot2” package in R (Wickham, 2016), and all the analyses were performed in R version 3.5.2 (R Core Team, 2019).

#### Assumption Check

Two key assumptions need to be met to apply the GRM:

##### Unidimensionality

A single latent trait variable should account for a large proportion of the common variance among item responses (Embretson & Reise, 2013). To check this assumption, we performed an exploratory factor analysis (EFA) with the package “psych” in R (Revelle, 2018), using the “fa” function. We extracted one factor, using minimal residuals method. As a rule-of-thumb, a factor should account for at least 20% of the variance for the questionnaire to meet the assumption and to obtain stable parameter estimates in the IRT model (Reckase, 1979). To explore the robustness of this assumption, we performed an exploratory bifactor analysis, using the “omega” function in the “psych” package with the minimal residuals’ method. Discrepancies among the general factor loadings in the bifactor model and the loadings in the unidimensional model are an indication of problems with unidimentionality (Reise et al., 2010).We compared the loadings of the general factor in both analyses to identify potential discrepancies.

##### Local independence

The probability of reporting a symptom in the questionnaire is strictly determined by the participant’s level of severity of FSS; hence, items are independent of one another, conditional on the level of severity of FSS (Embretson & Reise, 2013). To check this assumption, we used the “residuals” function of the “mirt” package (Chalmers, 2012). We calculated the Cramer’s V effect sizes for each item using four degrees of freedom. Cramer’s V calculates goodness of fit to indicate if data are independent of each other. A small (≤.05) to medium (≤.15) Cramer’s V effect size is interpreted as weak evidence against the local independence assumption (Cohen, 2013).

Additionally, we checked the fit of the items to the GRM model with the Kang and Chen’s signed chi-square test (S-χ^2^) using the “mirt” package (Chalmers, 2012), and the monotonicity using the “mokken” package (Van der Ark, 2007) in R.

### Sensitivity Analyses

We reproduced the statistical analyses using the data of all the adult participants from the Lifelines cohort who completed the SCL-90 somatization subscale (N = 148,604), to assess the comparability of the results.

For confidentiality reasons, the data used for this study is not available. The analyses code, results, and appendices are available on the Open Science Framework (https://osf.io/nj9as/).

## Results

### Sample Descriptive Statistics

**Figure 1.**
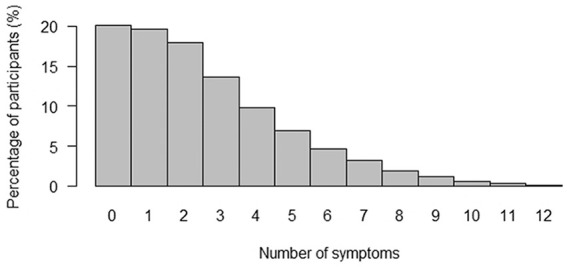
Number of symptoms reported. *Note*. Response to items of the SCL-90 somatization scale were dichotomized into absent (answer option 0), and present (answer options 1, 2, 3, and 4) to calculate the symptom count.

From the 82,740 participants, 47% were male and 53% were female. The mean age was 42 years (*SD* = 12, min = 18, max = 90). When calculating the mean item scores (adding the score of the 12 items and dividing by the number of items), the median score of the SCL-90 somatization scale was .17 (interquartile range = .33, min = 0, max = 4.0). Figure 1 shows that, when calculating individual symptom count, 80% of the participants reported having at least one symptom of the SCL-90 somatization scale in the past 7 days.

## Distribution of Response Choices

Table 1 shows the proportion of responses of each answer option per SCL-90 somatization scale item. The answer option *not at all* (0) obtained 78% of the answers on average in all the items and the answer option *extremely* (4) obtained less than 1%, resulting in an extremely skewed distribution.

**Table 1.** Distribution of Response Choices per Item of the SCL-90 Somatization Scale in a Sample of Participants Without Somatic Conditions.

No.	Item	*Not at all* (0)	*A bit* (1)	*Moderately* (2)	*Quite a bit* (3)	*Extremely* (4)	NA
1	Headache	63.5%	30.6%	3.7%	1.8%	0.4%	0.1%
2	Dizziness	85.8%	12.2%	1.2%	0.5%	0.1%	0.2%
3	Pain in the chest or around the heart	92.4%	6.2%	0.7%	0.2%	0.0%	0.4%
4	Pain in the lower back	58.2%	30.3%	6.7%	3.5%	1.1%	0.3%
5	Nausea or upset stomach	81.9%	14.3%	2.4%	0.9%	0.3%	0.2%
6	Painful muscles	57.0%	31.8%	6.9%	3.1%	1.0%	0.2%
7	Difficulty breathing	93.7%	5.2%	0.6%	0.2%	0.0%	0.2%
8	Feeling alternately hot and cold	81.7%	13.4%	3.1%	1.3%	0.4%	0.1%
9	A numb or tingling feeling in some body part	81.8%	13.2%	2.9%	1.5%	0.5%	0.2%
10	A lump in your throat	88.6%	9.0%	1.5%	0.6%	0.2%	0.2%
11	Feeling weak physically	76.8%	18.3%	3.2%	1.1%	0.4%	0.2%
12	Heavy feelings in arms or legs	78.2%	17.1%	3.0%	1.2%	0.4%	0.1%

*Note*. SCL-90 = Symptom Checklist–90; NA = Not available (missing).

### Assumption Check

The EFA with a one-factor solution explained 23% of the total variance. The loadings on the general factor from the bifactor analysis were comparable with those from the EFA (see Appendix B in the online supplemental material). Thus, there is evidence supporting the unidimensional assumption. Regarding local independence, all items had small Cramer’s V effect sizes (between −0.04 and 0.07), which suggests that the items are independent enough from each other (see Appendix C in the online supplemental material). All items showed goodness of fit according to the root mean square error of approximation (<0.06) of the S-χ^2^ test. There were no indications of violation to the monotonicity, with acceptable item-scalability coefficients (H between 0.21 and 0.36).

### The Graded Response Model Parameters

#### Item Parameters

Table 2 shows the estimated item parameters from the GRM. The items of the somatization scale from the SCL-90 are listed in increasing order of mean threshold value, that is, from the least to the most severe. As shown in Table 2, most item threshold parameters have large values, indicating that a high level of severity of FSS is necessary to report the highest answer options of an item (i.e., *quite a bit* or *extremely*). This is due to the large proportion of the participants reporting *not at all* (0) in all items, reducing the probability of choosing higher answer options and inflating the threshold parameters. Regarding the discrimination parameter (α), Item 12 (heavy feelings in arms or legs) was the best at discriminating between levels of severity of FSS, followed by Item 11 (feeling weak physically). These two items had low mean thresholds, meaning that they were reported at lower severity levels of FSS. Items 1 (headache) and 4 (pain in the lower back) showed the lowest discrimination, indicating that these items were the least able to distinguish between persons with different levels of severity of FSS.

**Table 2.** IRT Parameters for Each Item in the Subsample Without Somatic Conditions.

No.	Item	Discrimination parameter (α)	Threshold parameters (β)				Mean threshold
			β1	β2	β3	β4	
6	Painful muscles	1.37	0.27	1.94	2.87	4.06	2.28
12	Heavy feelings in arms or legs	**2.50**	0.94	2.07	2.70	3.44	2.29
11	Feeling weak physically	**2.33**	0.90	2.10	2.80	3.53	2.33
4	Pain in the lower back	0.99	0.40	2.41	3.48	5.07	2.84
8	Feeling alternately hot and cold	1.39	1.43	2.71	3.57	4.66	3.09
9	A numb or tingling feeling in some body part	1.29	1.50	2.83	3.64	4.82	3.20
1	Headache	0.96	0.68	3.29	4.45	6.37	3.69
5	Nausea or upset stomach	1.16	1.62	3.33	4.37	5.58	3.72
2	Dizziness	1.32	1.76	3.64	4.53	5.82	3.94
10	A lump in tour throat	1.13	2.20	3.82	4.86	6.22	4.28
7	Difficulty breathing	1.40	2.50	4.08	5.12	6.46	4.54
3	Pain in the chest or around the heart	1.14	2.67	4.56	5.76	7.40	5.10

*Note*. Numbers in bold represent items with the highest discrimination parameter (α). IRT = item response theory.

#### Person Parameters

Regarding person location (θ), 28.4% of the sample had a score of −1; 43.1% had a score of 0; 23.3% had a score of 1; and 5.2% had a score of 2 or more.

### Category Response Curves

**Figure 2.**
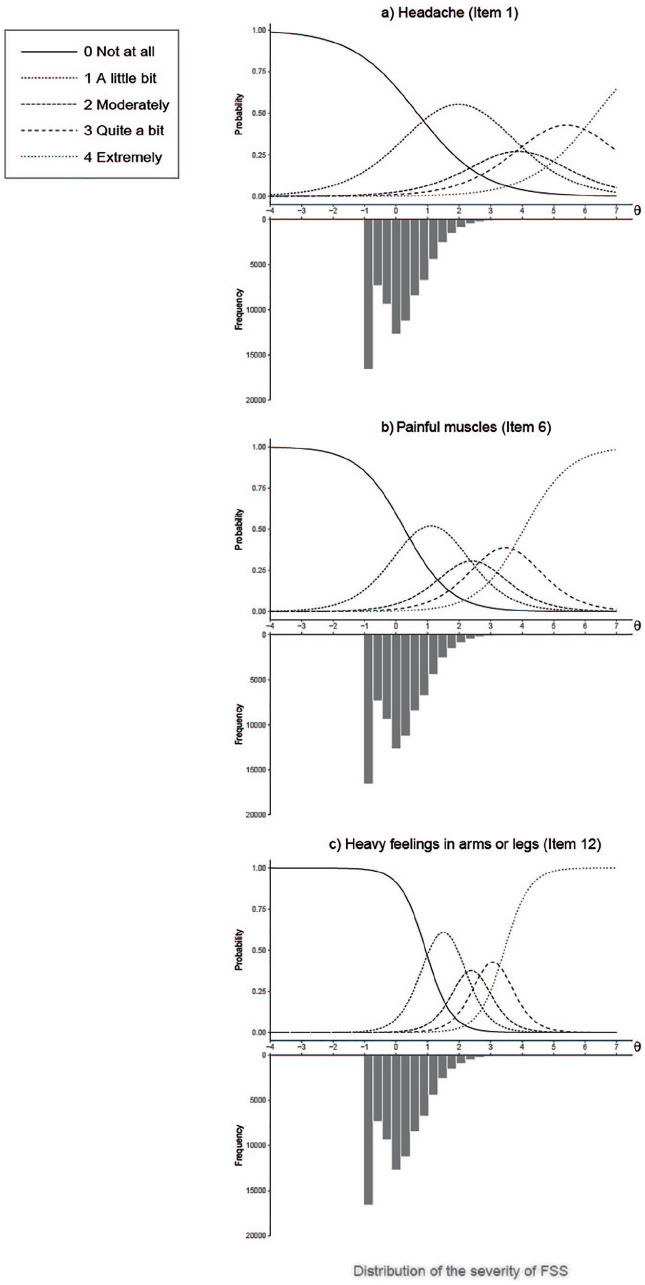
Category response curves (CRCs).

Figure 2 shows the CRCs of Items 1 (headache), 6 (painful muscles), and 12 (heavy feelings in arms or legs), which had the lowest, medium, and highest discrimination parameters, respectively. The top of each panel shows the CRCs and the bottom shows the distribution of the levels of severity of FSS (θ), where θ = 0 represents the mean level of severity of FSS. In this way, we can visualize what levels of severity of FSS increase the probability to choose an answer option of an item. IRT models are able to place both items and persons on a common scale, which is a distinct feature that is not shared with other measurement models. Note that the distribution of θ remains constant across items since it was calculated based on the whole questionnaire.

As shown in figure 2, in Item 1 (headache) the probabilities of choosing answer option “a little bit” peak around θ = 2, and this score increases for other answer options. Thus, less than 5.2% of the participants will report the highest answer options in this item. The CRCs of this item are wide and present low peaks, reflecting that the item is not able to discriminate well between different levels of severity of FSS. In Item 6 (Painful muscles), the answer options peak at a lower θ (e.g., the probabilities of choosing answer option “a little bit” peak around θ = 1), which means that this item is better at discriminating patients at a lower level of severity of FSS, as also shown in Table 2, where this item has the lowest mean threshold. The CRCs are narrower and more peaked, showing a better discrimination between different levels of FSS. Item 12 (heavy feelings in arms or legs), shows the highest discrimination parameter, as can be seen by its peaked and narrow CRCs. This means that this item is very good at distinguishing among different levels of severity of FSS. The CRCs from all the items of the SCL-90 somatization scale are presented in Appendix D (available in the online supplemental material).

### Item Information Functions

**Figure 3.**
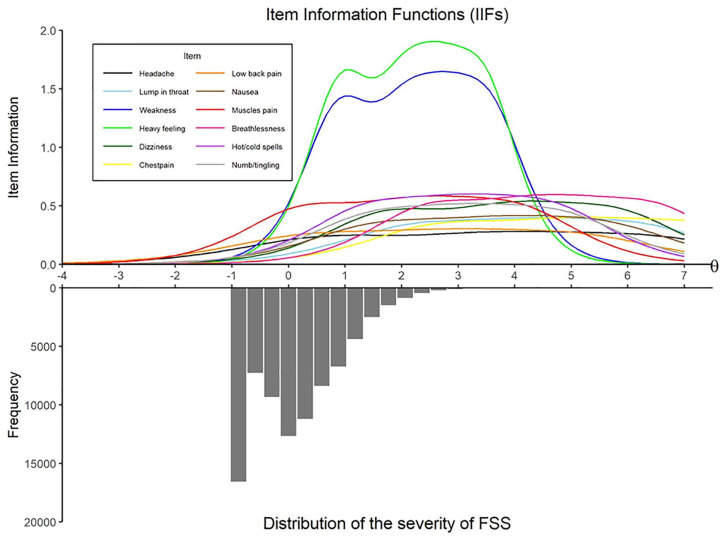
Item information functions. *Note*. FSS = functional somatic symptoms.

Figure 3 shows the IIF of each of the 12 items of the SCL-90 somatization subscale. Items 11 (feeling weak physically) and 12 (heavy feelings in arms or legs) provide the most information, thus, the *SE* is lower for these items. These two items provide the most precise measurement of the level of severity of FSS compared with the rest of the SCL-90 somatization items for individuals with θ between 0 and 4, that is, for the top 72% of the sample.

### Test Information Function

**Figure 4.**
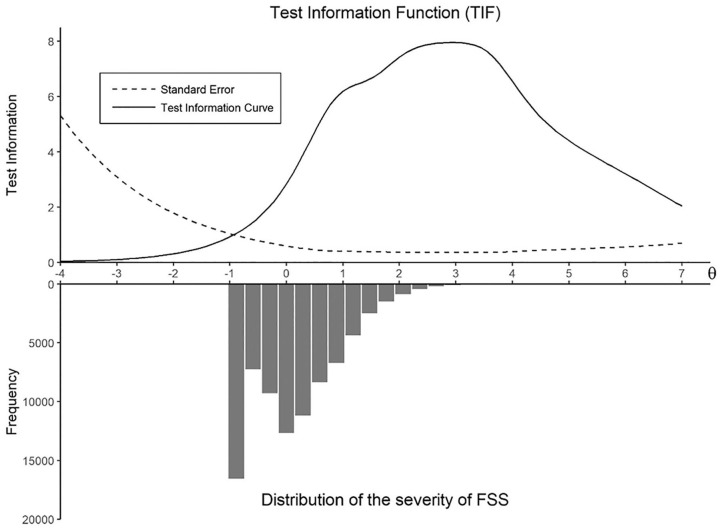
Test information function. *Note*. FSS = functional somatic symptoms.

Figure 4 shows the TIF and the *SE* of the scale. When the TIF increases, the *SE* decreases. The scale overall provides a maximum amount of information at the 99th percentile of severity of FSS, and lower *SE* when individuals score a θ between 1 and 4, that is, the top 28% of the sample.

### Sensitivity Analyses

The results of the analysis with all the participants who completed the somatization scale of the SCL-90 at baseline (*N* = 148,604), were highly comparable to the main results. There was no strong evidence of violation of the assumptions of unidimensionality and local independence. We obtained the same order of the items by mean threshold as in the main analyses, with mean thresholds varying from 1.84 to 4.54, which are slightly lower compared with the subsample without somatic conditions. Items 11 (feeling weak physically) and 12 (heavy feelings in arms or legs) had the highest discrimination estimates. Regarding the IIFs, Items 11 and 12 provided the most information. A full report of these analyses is available in https://osf.io/y3m5h/.

## Discussion

This study aimed to identify which of the SCL-90 somatization scale items most accurately reflected varying levels of severity of FSS and better discriminated between different levels of severity of FSS, in a sample of participants without somatic conditions. To this end, the GRM was fitted to the data. This allows to gain insight into the individual items of the SCL-90 somatization scale, as well as to determine the location of each person on the severity of the FSS latent scale.

Overall, high levels of severity of FSS were necessary for a person to report the higher answer options on the items of the SCL-90 somatization scale, which was reflected in the high threshold parameters (β). However, this is typically observed with clinical instruments (Reise & Waller, 2009), and it is expected given the nature of our sample (i.e., a population sample without somatic conditions). On average, the answer option *not at all* (0) received 78% of the answers for each item in our data set, which resulted in a very low number of participants with high levels of severity of FSS (i.e., only 4.6% of participants obtained a θ ≥ 2, and less than 1% obtained a θ ≥ 3), as can be seen in its distribution (Figures 2-4). This is reflected in the CRCs, where the curves of the answer option *not at all* (0) displace the rest of the curves, to be able to account for a large part of the lower person location scores (θ). The mean threshold parameters ranged from 2.3 to 5.1, suggesting that the items are best at measuring participants who have at least a level of severity of FSS (θ) higher than 2, that is, the top 5.2% of the sample.

The mean threshold parameters, used as a measure of the location of the items in a continuum of severity of FSS, were the lowest for Items 6 (painful muscles), 12 (heavy feelings in arms or legs), and 11 (feeling weak physically). These items reflect a lower level of severity of FSS compared with the rest of the items. In contrast, Items 10 (A lump in your throat), 7 (difficulty breathing), and 3 (pain in the chest or around the heart) reflected the highest levels of severity of FSS. Theoretically, these results may imply that items related to fatigue and nonspecific bodily symptoms are reported more frequently, and are thus, less severe, whereas items related to symptoms of specific body parts, such as pain in the chest, are rarely reported, and are more severe. Clinically, these results mean that a person who reports the most severe items may require more attention than a person who reports less severe items, even when these persons have the same sum score in the SCL-90 somatization scale.

Regarding item discrimination, Items 11 (feeling weak physically) and 12 (heavy feelings in arms or legs) were the most discriminative among different levels of severity of FSS, obtaining the highest discrimination parameters (α) and showing the most peaked slopes in the CRCs. Moreover, these items were the most informative about the levels of severity of FSS compared with the rest of the items, as shown in the IIFs. Thus, these two items show the highest precision at measuring the severity FSS in a sample of participants without somatic conditions. These findings are consistent with previous studies from clinical samples. A study in 3,078 patients with psychiatric disorders, admitted in day hospitals, reported the highest discrimination parameters for Item 11 (α = 2.80), and Item 12 (α = 2.62), on a selection of six items from the somatization scale of the SCL-90-R (Paap et al., 2011). Another study in 10,920 patients with neuromuscular diagnoses also reported high discrimination parameters for Item 11 (α = 1.36) and Item 12 (α = 1.25) compared with other items, on a selection of 17 items from the SCL-90- R measuring depression and somatization (C. L. Hart et al., 2003). The results of these studies show that these two items represent high discrimination parameters in clinical populations. Our study shows that these two items are also good at discriminating in a nonclinical population in which we assume that symptoms are not due to somatic conditions. Furthermore, the results of the sensitivity analyses of the entire sample, including participants with and without somatic conditions, were highly comparable with the results of our main analysis.

It is worthwhile to highlight that Items 11 and 12 had the highest discrimination parameters and also represented lower severity of FSS. This could be due to the low levels of severity of FSS in the sample. These items allow to better discriminate between subjects in the range of low severity of FSS in comparison with the remaining items.

Consistencies between previous results and ours indicate that Items 11 and 12 are the most appropriate to measure and discriminate the levels of severity of FSS in various populations. This is especially relevant since previous studies have highlighted the need to better select the items that should be included in the questionnaires to measure FSS (van Driel et al., 2017; Zijlema et al., 2013). The SCL-90, as well as other instruments measuring FSS, have been constructed based on experts’ knowledge (Derogatis, 1994; Zijlema et al., 2013). Previous studies highlighted headaches, nausea, shortness of breath, dizziness and low back pain as the most relevant FSS symptoms according to the experts (Kroenke & Mangelsdorff, 1989; Zijlema et al., 2013), however our study showed that headache and low back pain have the lowest discrimination parameters and provide little information about the level of severity of FSS. Thus, the results of our study indicate that there are inconsistencies between experts’ opinion and empirical evidence. Therefore, a data-driven approach to select the items to measure FSS could be beneficial for the construction of shorter and more accurate questionnaires and could help clinicians identify the severity of FSS in individuals more precisely.

The present study has several strengths, including the large population-based cohort, and the comparability of our findings to the population cohort including participants with somatic conditions. Moreover, the use of sophisticated IRT methods, such as the GRM, provides insights on the characteristics of individual symptoms at measuring FSS by ordering symptoms on a continuum of severity of FSS, instead of treating them as counts for a diagnostic threshold (Aggen et al., 2005; Reise & Waller, 2009). Another strength is the quality of the instrument chosen for analysis. The SCL-90 somatization scale has been highlighted as one of the most suitable instruments to measure FSS in large scale studies given its psychometric properties, the inclusion of relevant symptoms, and the response options assessing symptom severity (Zijlema et al., 2013). Although previous studies have used IRT methods on the SCL-90, none has aimed to explore the severity and discrimination of the items of the somatization scale in a general population sample or a sample without somatic conditions. This can be especially informative for clinicians given that persons presenting with FSS typically report their symptoms in primary care settings.

While interpreting the results, several limitations should be considered. First, we used self-reported measures for excluding the participants with somatic conditions, which potentially leads to selection bias since there is not a certain diagnostic of their condition. Second, by excluding somatic conditions, we aimed to have a population whose symptoms were not explained by a somatic condition. However, symptoms could have been caused by other somatic conditions/illnesses that we did not exclude. Thus, it is uncertain if the symptoms reported in the SCL-90 questionnaire are FSS. Nevertheless, we found comparable results in our sensitivity analyses, thus we do not expect that the presence of somatic conditions has a large influence on our results. Third, during the translation to Dutch language, Item 11 (feeling weak physically) was translated with a slightly different meaning from the original SCL-90 item (feeling weak in parts of your body). Given that this item had one of the highest discrimination parameters, this result may not be generalizable to the original SCL-90 somatization subscale; however, previous studies with the original item have shown similar results to the ones found in our study. This implies that differences in terms used in the item had minimal influence on the results, and that “feeling weak” in general, is a relevant symptom to assess the severity of FSS. Finally, although most participants reported one or more symptoms (Figure 1), their severity was low in our presumably healthy population. It would be interesting to see whether our results would be consistent in a population with high levels of FSS.

Considering the results of this study, research on the measurement methods for FSS could continue to benefit from IRT. It would be worthwhile to perform the analysis from the current study in a population of participants whose symptoms have been objectively identified as medically unexplained. On the other hand, the somatization scale of the SCL-90 has provided relevant information on which items are the most discriminative of severity of FSS, however, this scale has only 12 symptoms. Therefore, it would be worthwhile to perform this analysis in questionnaires including a wider collection of symptoms, in order to identify relevant items for the measurement of FSS. This could provide even more insight towards the construction of more accurate questionnaires.

In conclusion, we identified two items that can best discriminate between levels of severity of FSS in a population without somatic conditions; namely, “feeling weak physically” and “heavy feelings in arms or legs.” These results were consistent with previous studies in clinical samples, and with our sensitivity analyses including all participants from a population-based cohort. Clinicians and researchers may pay extra attention to these symptoms to augment the assessment of FSS.

## Supplemental Material

Supplement_material – Supplemental material for Improving the Measurement of Functional Somatic Symptoms With Item Response Theory Click here for additional data file. Supplemental material, Supplement_material for Improving the Measurement of Functional Somatic Symptoms With Item Response Theory by Angélica Acevedo-Mesa, Jorge Nunes Tendeiro, Annelieke Roest, Judith G. M. Rosmalen and Rei Monden in Assessment
