# Supplementary material for: Improving the Measurement of Functional Somatic Symptoms With Item Response Theory
Source: Assessment. 2020 Aug 6;28(8):1960–70. doi: 10.1177/1073191120947153 (PMC8543564; doi:10.1177/1073191120947153)
Supplement: Supplement_material – Supplemental material for Improving the Measurement of Functional Somatic Symptoms With Item Response Theory [file Supplement_material.pdf]

# Appendix A

## Table S1. Excluded diseases.

| Disease | Count |
|---|---|
| Heart attack | 1,469 |
| Arrhythmia | 11,180 |
| Heart failure | 1,047 |
| Diabetes type 1 or 2 | 3,555 |
| Stroke | 1,038 |
| Osteoarthritis | 11,021 |
| COPD | 7,826 |
| Dementia | 18 |
| Rheumatoid arthritis | 3,086 |
| Asthma | 13,223 |
| Cancer (any) | 6,679 |
| Parkinson | 79 |
| Epilepsy | 1,842 |
| Disturbed kidney function | 3,776 |
| Migraine | 27,442 |
| Osteoporosis | 2,206 |
| Arthrosis | 0 |

# Appendix B

**Table S2. Exploratory Factor Analysis (EFA).**

| Item | Loading |
|---|---|
| Headache | 0.42 |
| Dizziness | 0.45 |
| Pain in the chest or around the heart | 0.34 |
| Pain in the lower back | 0.42 |
| Nausea or upset stomach | 0.43 |
| Painful muscles | 0.54 |
| Difficulty breathing | 0.38 |
| Feeling alternately hot and cold | 0.48 |
| A numb or tingling feeling in some body part | 0.46 |
| A lump in your throat | 0.37 |
| Feeling weak physically | 0.69 |
| Heavy feelings in arms or legs | 0.69 |
| **Proportion variance 0.23** | |

**Table S3. Bifactor Analysis.**

| **Items** | **General factor** | **F1*** | **F2*** | **F3*** |
|---|---|---|---|---|
| Headache | 0.33 | 0.30 | | |
| Dizziness | 0.35 | 0.36 | | |
| Pain in the chest or around the heart | 0.26 | 0.28 | | |
| Pain in the lower back | 0.39 | | 0.43 | |
| Nausea or upset stomach | 0.33 | 0.35 | | |
| Painful muscles | 0.53 | | 0.44 | |
| Difficulty breathing | 0.30 | 0.30 | | |
| Feeling alternately hot and cold | 0.40 | 0.26 | | |
| A numb or tingling feeling in some body part | 0.42 | | 0.22 | |
| A lump in your throat | 0.29 | 0.26 | | |
| Feeling weak physically | 0.60 | 0.31 | | 0.24 |
| Heavy feelings in arms or legs | 0.74 | | | 0.49 |
| **Eigenvalues** | 2.25 | 0.75 | 0.44 | 0.33 |

## Appendix C

**Table S4. Local independence.**

| **Item** | *2 Dizziness* | *3 Pain in the chest or around the heart* | *4 Pain in the lower back* | *5 Nausea or upset stomach* | *6 Painful muscles* | *7 Difficulty breathing* | *8 Feeling alternately hot and cold* | *9 A numb or tingling feeling in some body part* | *10 A lump in your throat* | *11 Feeling weak physically* | *12 Heavy feelings in arms or legs* |
|---|---|---|---|---|---|---|---|---|---|---|---|
| *1 Headache* | 0.07 | 0.03 | 0.04 | 0.04 | -0.05 | -0.02 | 0.04 | -0.04 | 0.02 | -0.04 | -0.04 |
| *2 Dizziness* | | 0.03 | -0.02 | 0.04 | -0.04 | 0.03 | 0.02 | 0.02 | 0.02 | -0.03 | -0.03 |
| *3 Pain in the chest or around the heart* | | | -0.03 | 0.03 | -0.03 | 0.08 | -0.02 | 0.02 | 0.04 | -0.03 | -0.02 |
| *4 Pain in the lower back* | | | | -0.03 | 0.12 | -0.02 | -0.03 | 0.06 | -0.03 | -0.05 | -0.04 |
| *5 Nausea or upset stomach* | | | | | -0.04 | 0.02 | 0.04 | -0.03 | 0.03 | 0.05 | -0.04 |
| *6 Painful muscles* | | | | | | -0.02 | -0.04 | 0.07 | -0.03 | -0.06 | 0.06 |
| *7 Difficulty breathing* | | | | | | | 0.02 | -0.02 | 0.04 | 0.03 | -0.02 |

| | | | | |
|---|---|---|---|---|
| *8 Feeling alternately hot and cold* | -0.04 | 0.03 | -0.04 | -0.04 |
| *9 A numb or tingling feeling in some body part* | | -0.03 | -0.05 | 0.06 |
| *10 A lump in your throat* | | | 0.04 | -0.04 |
| *11 Feeling weak physically* | | | | 0.08 |

## Appendix D

**Figure S1. Category Response Curves (CRCs) Item 1 "Headache"**

**Figure S2. Category Response Curves (CRCs) Item 2 "Dizziness"**

**Figure S3. Category Response Curves (CRCs) Item 3 "Pain in the chest or around the heart"**

**Figure S4. Category Response Curves (CRCs) Item 4 "Pain in the lower back"**

**Figure S5. Category Response Curves (CRCs) Item 5 "Nausea or upset stomach"**

**Figure S6. Category Response Curves (CRCs) Item 6 "Painful muscles"**

**Figure S7. Category Response Curves (CRCs) Item 7 "Difficulty breathing"**

**Figure S8. Category Response Curves (CRCs) Item 8 "Feeling alternately hot ad cold"**

**Figure S9. Category Response Curves (CRCs) Item 9 "a numb or tingling feeling in some body part"**

**Figure S10. Category Response Curves (CRCs) Item 10 "A lump in your throat"**

**Figure S11. Category Response Curves (CRCs) Item 11 "Feeling weak physically"**

**Figure S12. Category Response Curves (CRCs) Item 12 "Heavy feelings in arms or legs"**

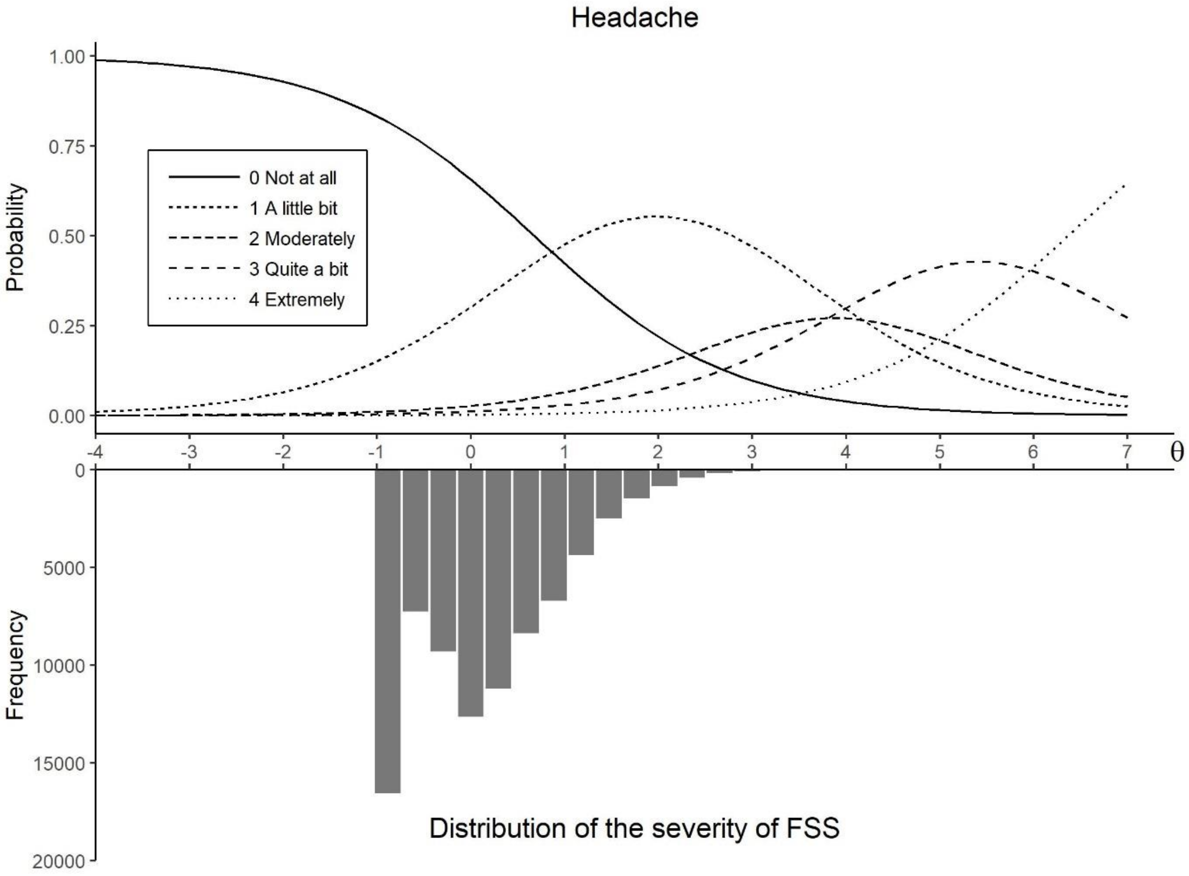
Headache
Probability
1.00
0.75
0.50
0.25
0.00
0 Not at all
1 A little bit
2 Moderately
3 Quite a bit
4 Extremely
-4
-3
-2
-1
0
1
2
3
4
5
6
7
θ
Frequency
0
5000
10000
15000
20000
Distribution of the severity of FSS

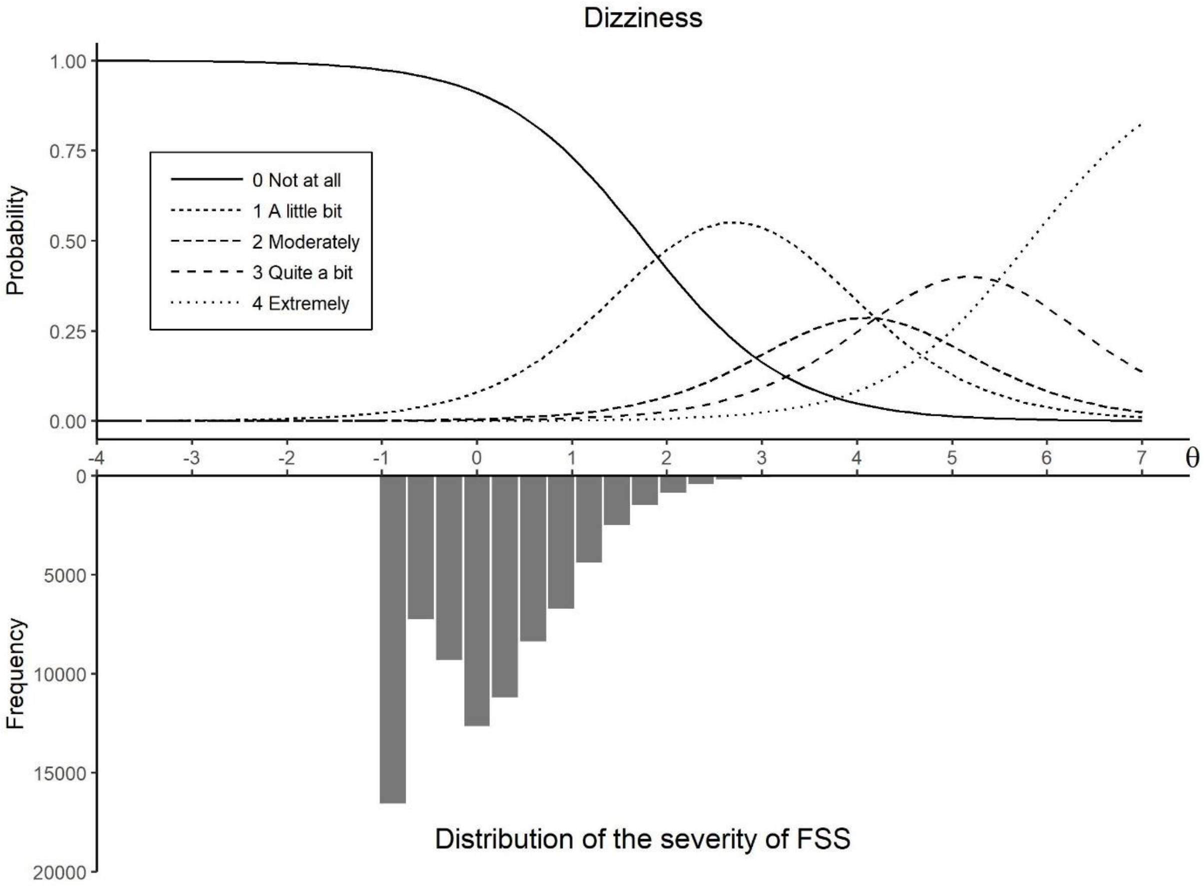
Dizziness
Probability
1.00
0.75
0.50
0.25
0.00
0 Not at all
1 A little bit
2 Moderately
3 Quite a bit
4 Extremely
-4
-3
-2
-1
0
1
2
3
4
5
6
7
θ
Frequency
0
5000
10000
15000
20000
Distribution of the severity of FSS

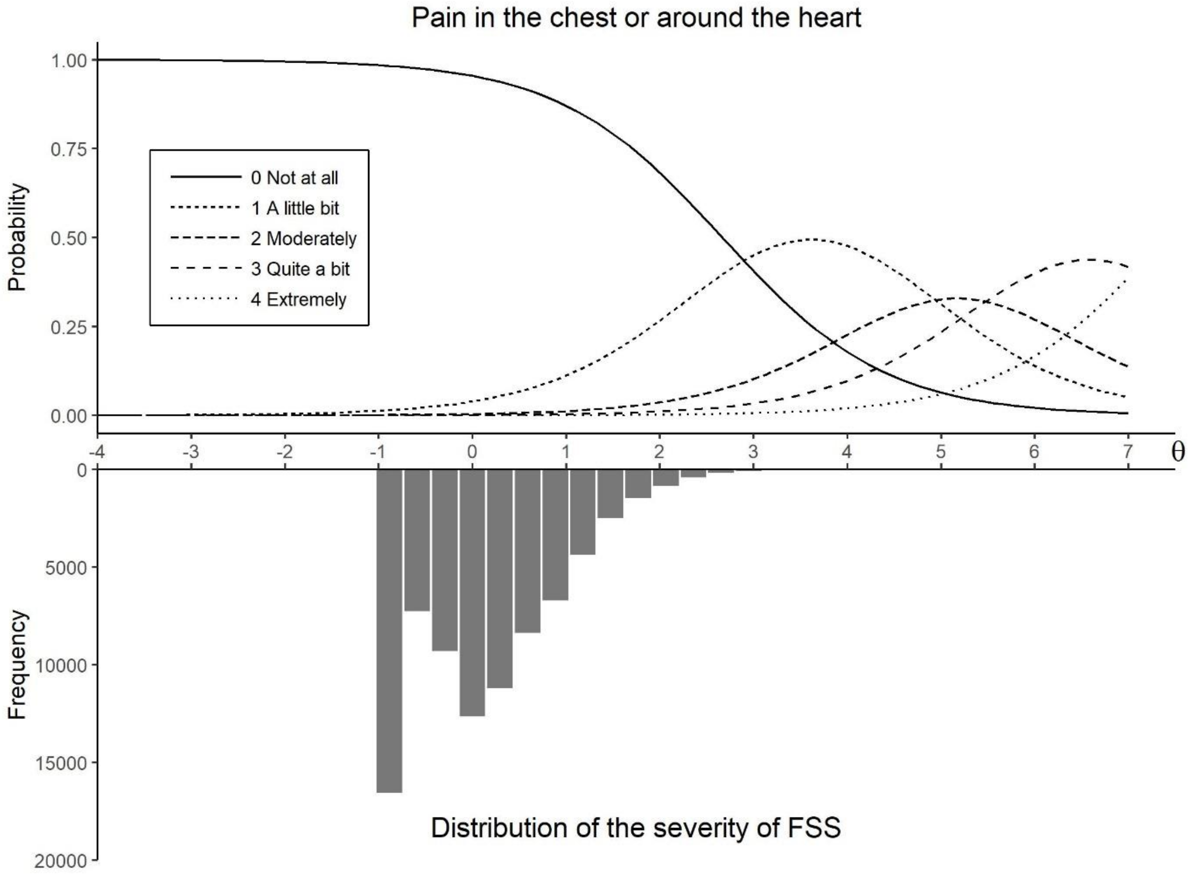

Pain in the chest or around the heart
Probability
1.00
0.75
0.50
0.25
0.00
0 Not at all
1 A little bit
2 Moderately
3 Quite a bit
4 Extremely
-4
-3
-2
-1
0
1
2
3
4
5
6
7
θ
Frequency
0
5000
10000
15000
20000
Distribution of the severity of FSS

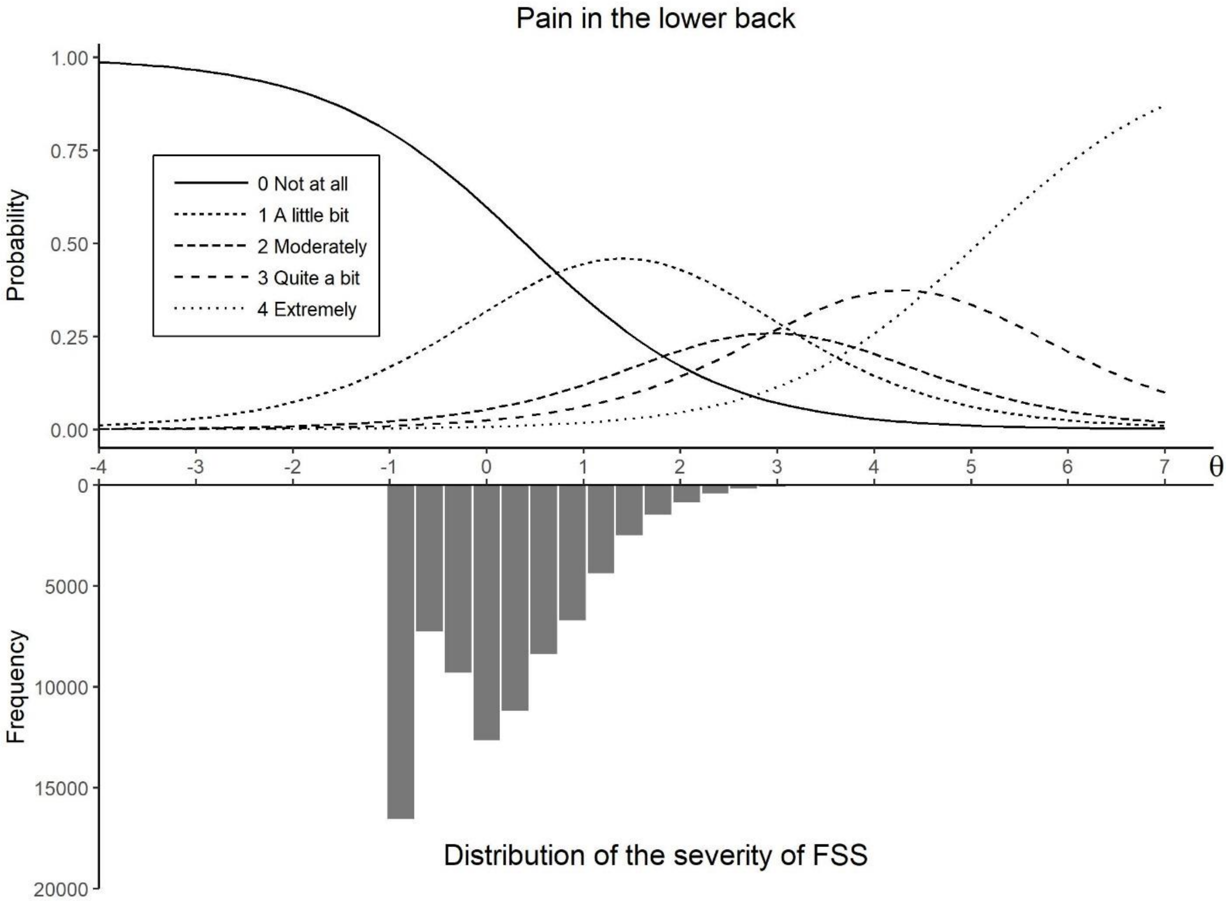
Pain in the lower back
Probability
1.00
0.75
0.50
0.25
0.00
0 Not at all
1 A little bit
2 Moderately
3 Quite a bit
4 Extremely
-4
-3
-2
-1
0
1
2
3
4
5
6
7
θ
Frequency
0
5000
10000
15000
20000
Distribution of the severity of FSS

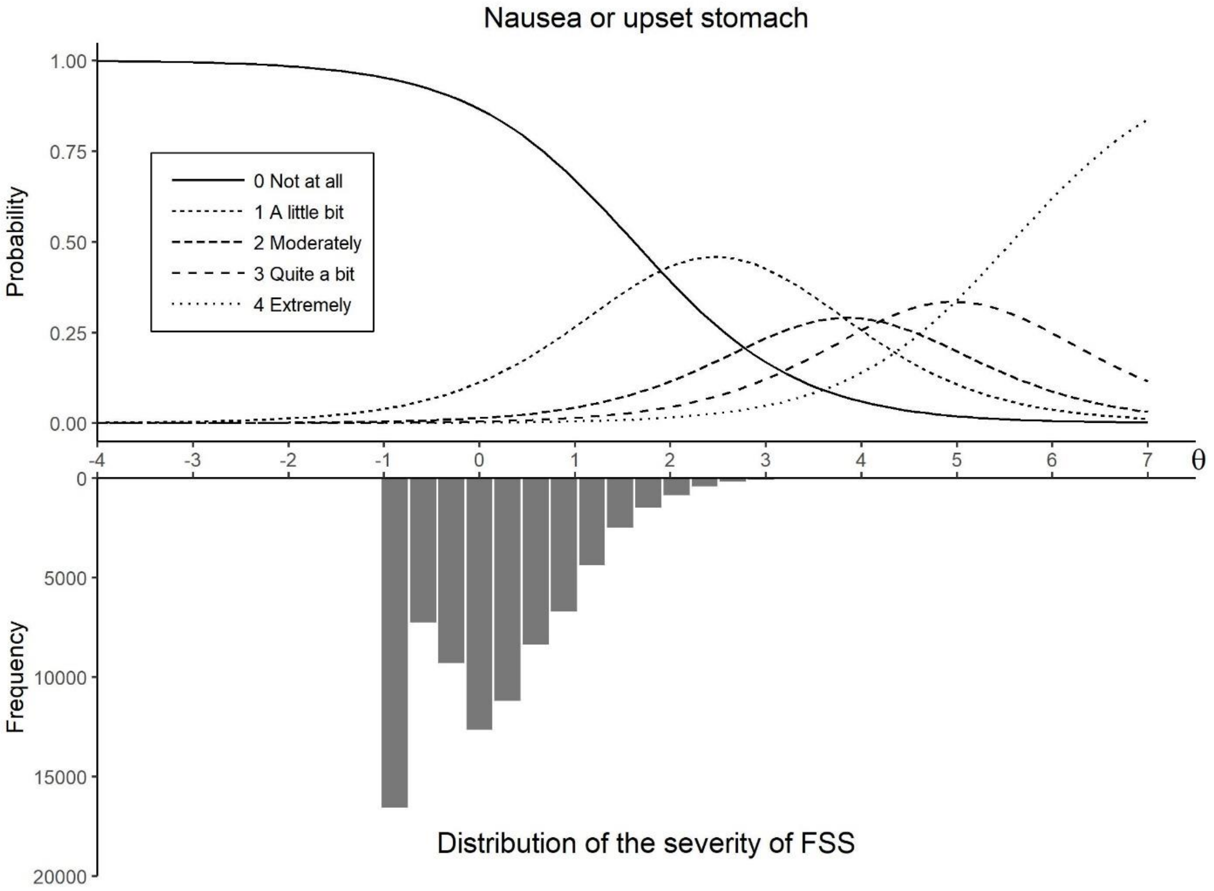
Nausea or upset stomach
Probability
1.00
0.75
0.50
0.25
0.00
0 Not at all
1 A little bit
2 Moderately
3 Quite a bit
4 Extremely
-4
-3
-2
-1
0
1
2
3
4
5
6
7
θ
Frequency
0
5000
10000
15000
20000
Distribution of the severity of FSS

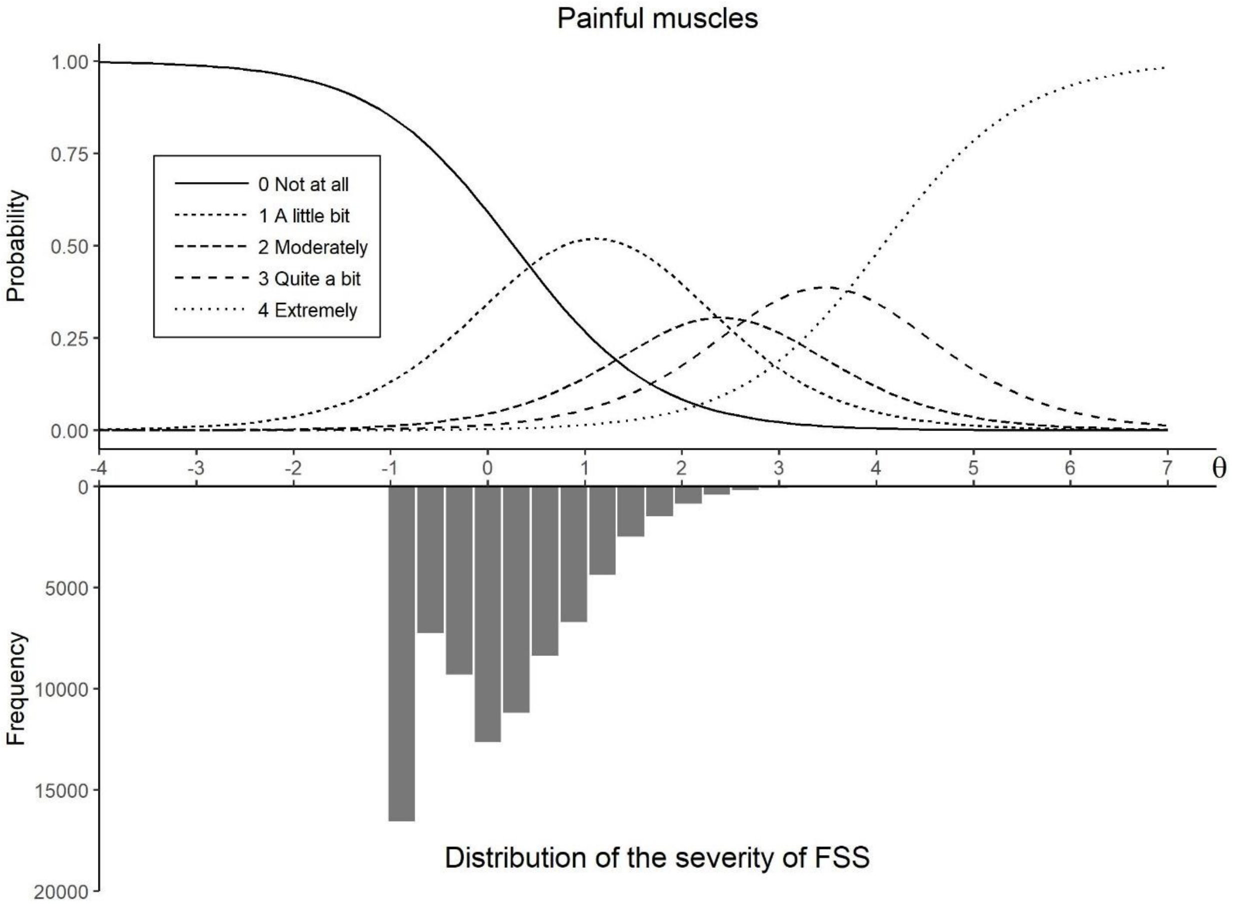

Painful muscles
Probability
1.00
0.75
0.50
0.25
0.00
0 Not at all
1 A little bit
2 Moderately
3 Quite a bit
4 Extremely
-4
-3
-2
-1
0
1
2
3
4
5
6
7
θ
Frequency
0
5000
10000
15000
20000
Distribution of the severity of FSS

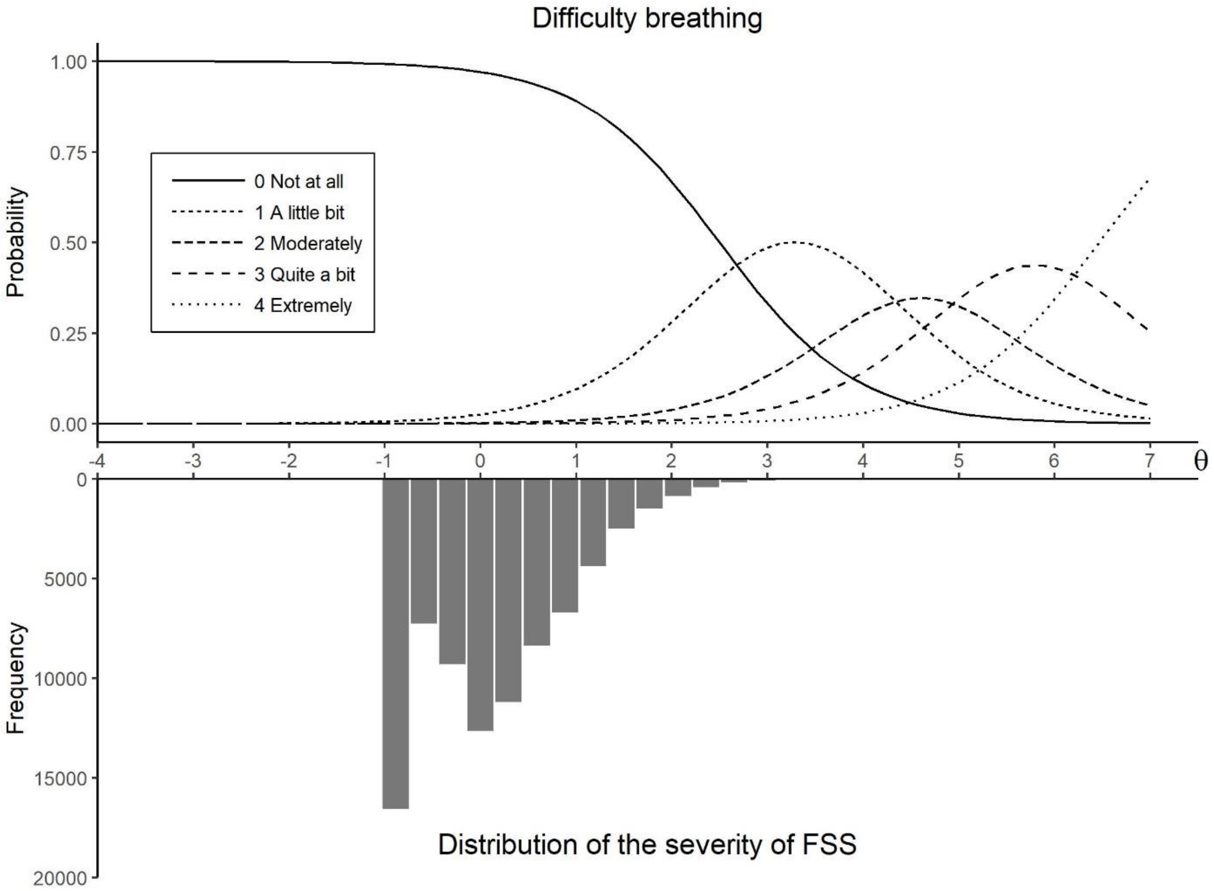
Difficulty breathing
1.00
0.75
0.50
0.25
0.00
Probability
0 Not at all
1 A little bit
2 Moderately
3 Quite a bit
4 Extremely
-4
-3
-2
-1
0
1
2
3
4
5
6
7
θ
0
5000
10000
15000
20000
Frequency
Distribution of the severity of FSS

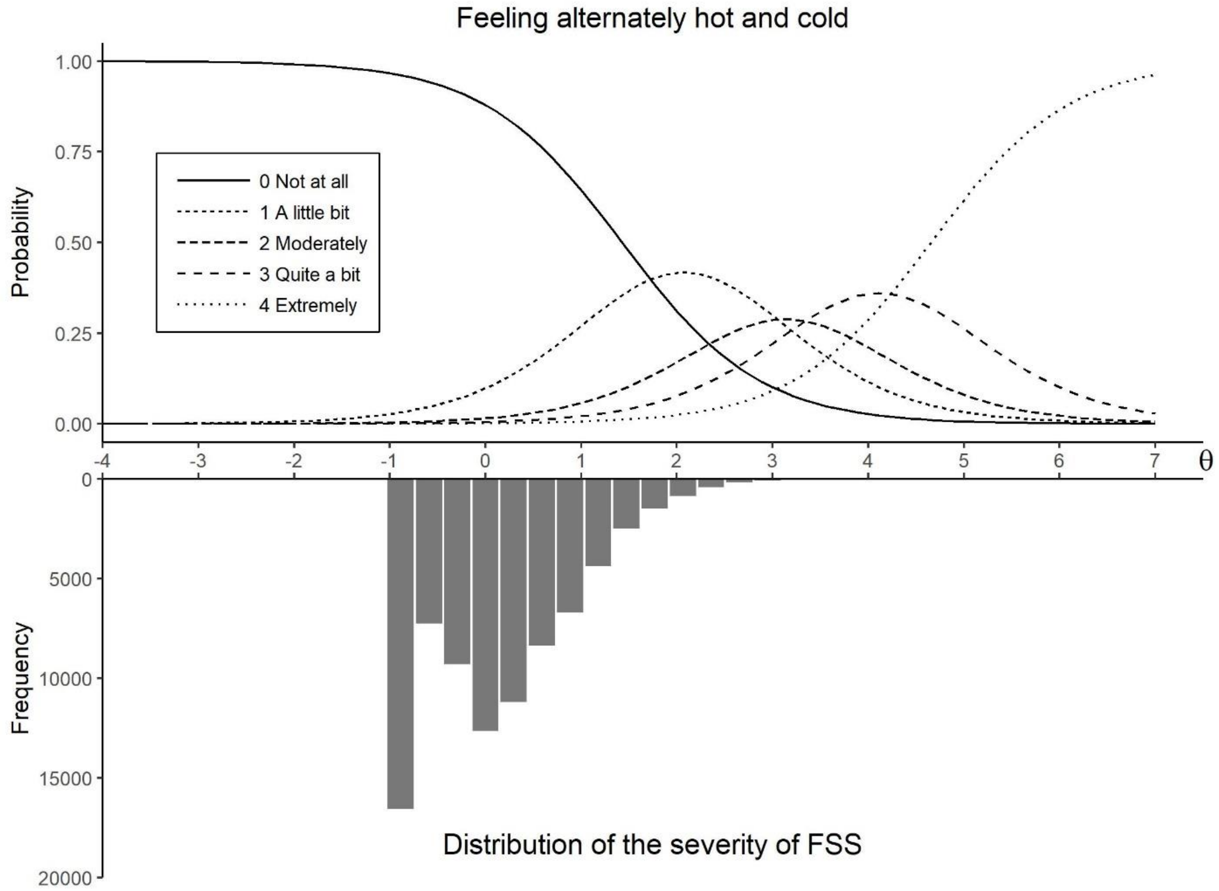
Feeling alternately hot and cold
1.00
0.75
0.50
0.25
0.00
Probability
0 Not at all
1 A little bit
2 Moderately
3 Quite a bit
4 Extremely
-4
-3
-2
-1
0
1
2
3
4
5
6
7
θ
0
5000
10000
15000
20000
Frequency
Distribution of the severity of FSS

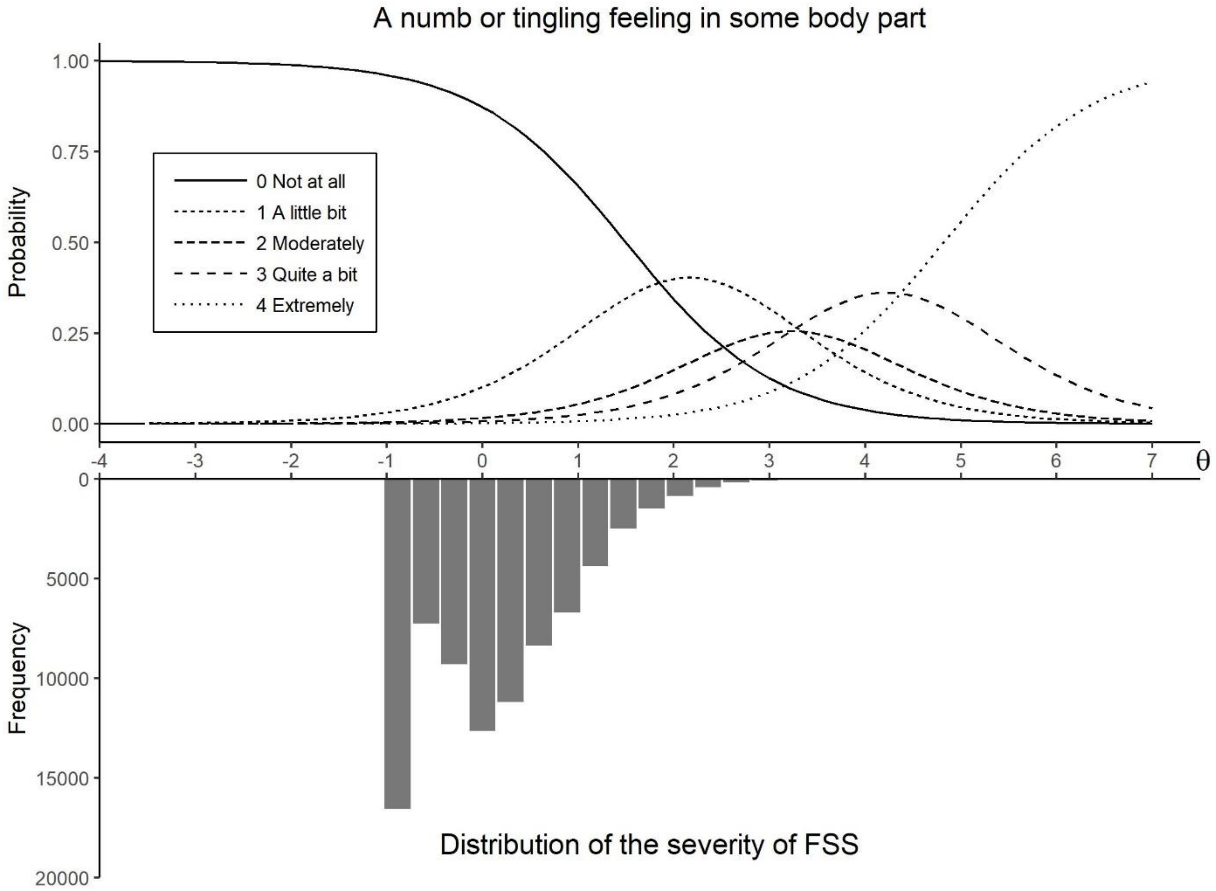
A numb or tingling feeling in some body part
Probability
1.00
0.75
0.50
0.25
0.00
0 Not at all
1 A little bit
2 Moderately
3 Quite a bit
4 Extremely
-4
-3
-2
-1
0
1
2
3
4
5
6
7
θ
Frequency
0
5000
10000
15000
20000
Distribution of the severity of FSS

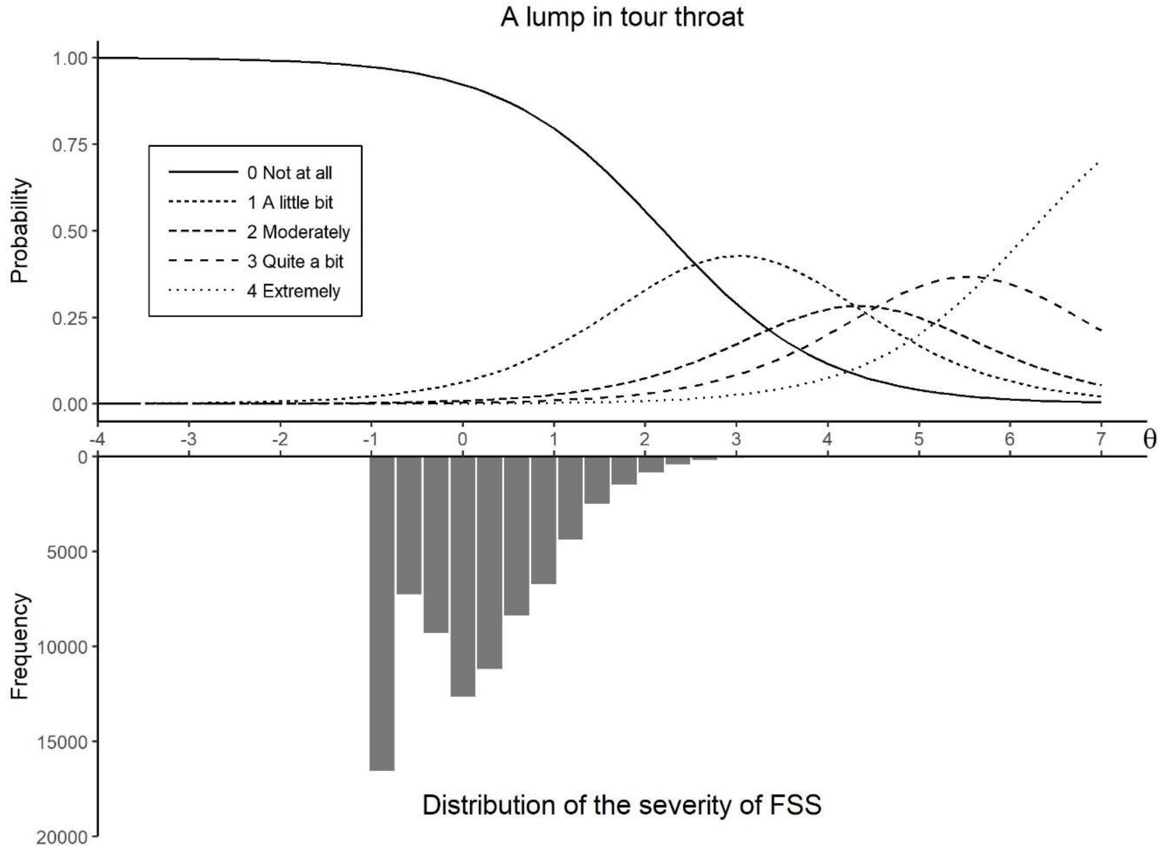
A lump in tour throat
Probability
1.00
0.75
0.50
0.25
0.00
0 Not at all
1 A little bit
2 Moderately
3 Quite a bit
4 Extremely
-4
-3
-2
-1
0
1
2
3
4
5
6
7
θ
0
5000
10000
15000
20000
Frequency
Distribution of the severity of FSS

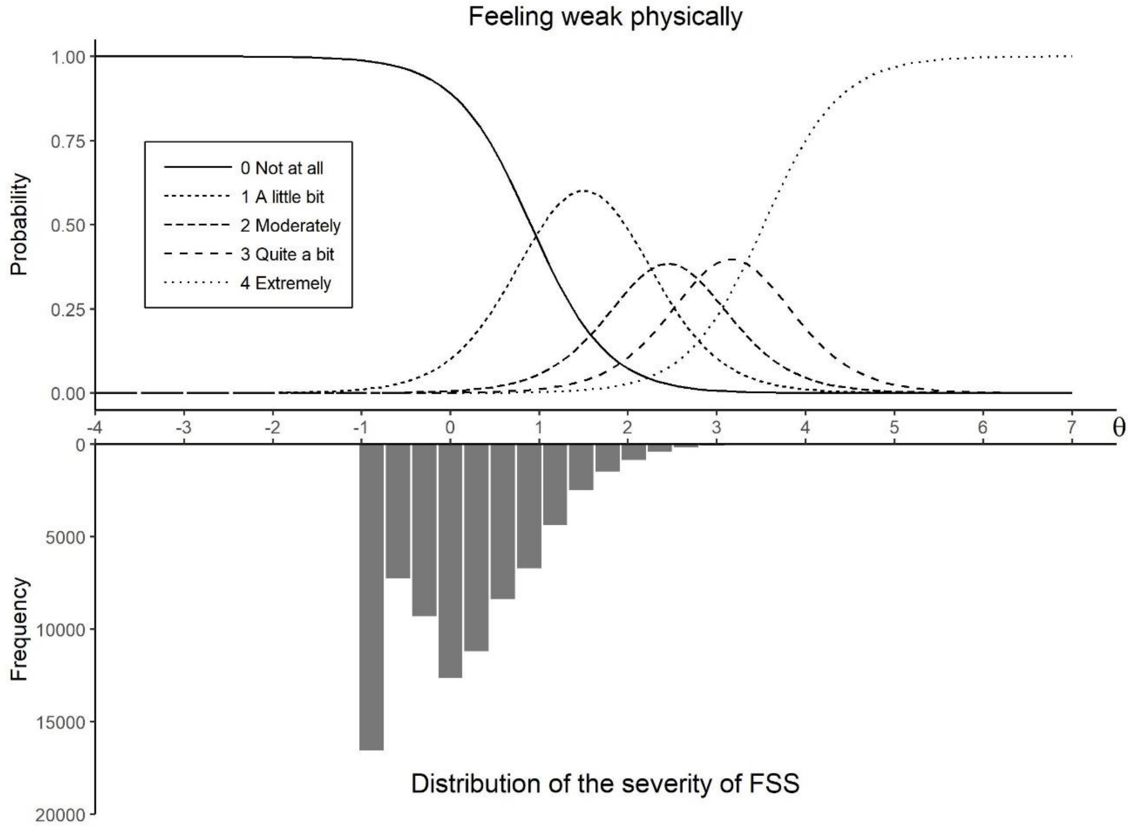

Feeling weak physically
Probability
1.00
0.75
0.50
0.25
0.00
0 Not at all
1 A little bit
2 Moderately
3 Quite a bit
4 Extremely
-4
-3
-2
-1
0
1
2
3
4
5
6
7
θ
Frequency
0
5000
10000
15000
20000
Distribution of the severity of FSS

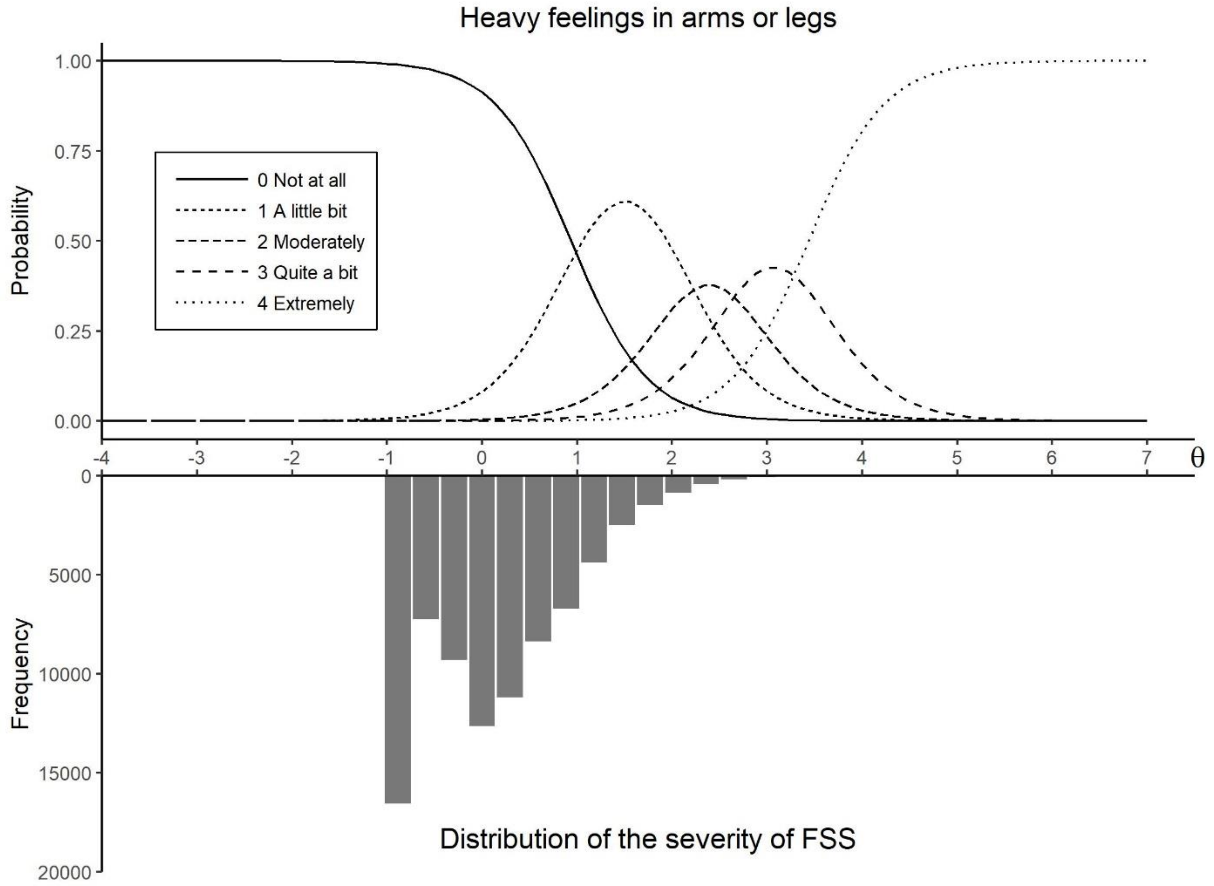
Heavy feelings in arms or legs
Probability
1.00
0.75
0.50
0.25
0.00
0 Not at all
1 A little bit
2 Moderately
3 Quite a bit
4 Extremely
-4
-3
-2
-1
0
1
2
3
4
5
6
7
θ
Frequency
0
5000
10000
15000
20000
Distribution of the severity of FSS
